# Supplementary material for: Acylpolyamine Mygalin as a TLR4 Antagonist Based on Molecular Docking and In Vitro Analyses
Source: Biomolecules. 2020 Dec 1;10(12):1624. doi: 10.3390/biom10121624 (PMC7761503; doi:10.3390/biom10121624)
Supplement: Supplementary file 1 [file biomolecules-10-01624-s001.pdf]

Table S1: Comparison of mygalin to similar drugs.

Drug	Smile	Similarity %	Database/Tools	Activity	Structure	Reference
N,N'-[1,12-Dodecanediylbis(imino-3,1-propanediyl)]bis(2,5-dimethoxybenzamide)	COc1ccc(OC)c(c1)C(=O)NCCCNCCCCCCCCCCCCNCCCNC(=O)c2cc(OC)ccc2OC	91.3	ChEMBL	antimalarial polyamines	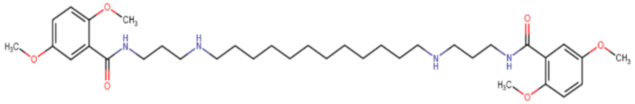	45
N,N'-1,8-Octanediylbis(2,3,4-trihydroxybenzamide)	Oc1ccc(C(=O)NCCCCCCCCNC(=O)c2ccc(O)c(O)c2O)c(O)c1O	88.9	ChEMBL	anti-HCV inhibitors.	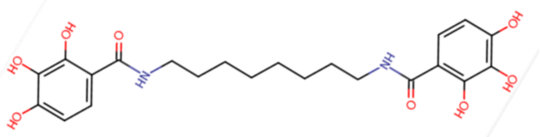	51
2-hydroxy-N-[6-[(2-hydroxy-5-methylbenzoyl)amino]hexyl]-5-methylbenzamide	Cc1ccc(O)c(c1)C(=O)NCCCCCCNC(=O)c2cc(C)ccc2O	88.7	ChEMBL	antibacterial activity	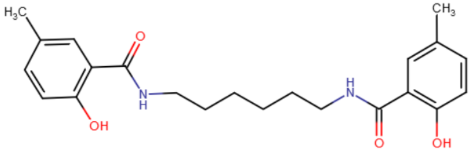	43
LCM (N,N'-butane-1,4-diylbis(2,3 dihydroxybenzamide)	O=C(c1cccc(c1O)O)NCCCCNC(=O)c1cccc(c1O)O	88.6	SwissSimilarity - Ligands from the PDB	siderophore	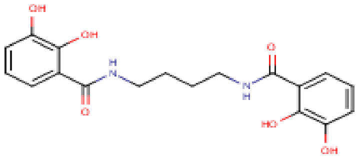	44 and PDB:5A1J
N,N'-1,5-Pentanediylbis(2,3-dihydroxybenzamide)	Oc1cccc(C(=O)NCCCCCNC(=O)c2cccc(O)c2O)c1O	86.0	ChEMBL	antimetastatic agents	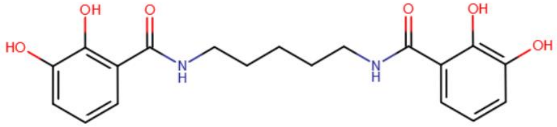	54
N-[3-[(2,3-Dihydroxybenzoyl)amino]propyl]-2,3-dihydroxybenzamide	Oc1cccc(C(=O)NCCCNC(=O)c2cccc(O)c2O)c1O	80.9	ChEMBL	HIV-1 integrase inhibitors	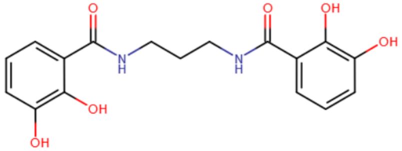	52

N,N'-1,6-Hexanediylbis(3,4,5-trihydroxybenzamide)	Oc1cc(cc(O)c1O)C(=O)NCCCCCCNC(=O)c2cc(O)c(O)c(O)c2	80.2	ChEMBL	inhibitors of catechol-*O*-methyltransferase (COMT)	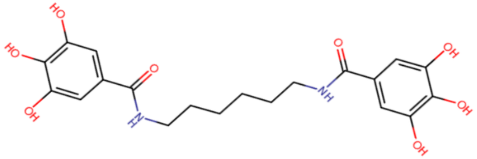	53
2,3-dihydroxy-N-[(5S)-6-hydroxy-5-[(3-hydroxybenzoyl)amino]hexyl]benzamide	OC[C@H](CCCCNC(=O)c1cccc(O)c1O)NC(=O)c2cccc(O)c2	79.8	ChEMBL	antiproliferative on leukemic K-562 cells	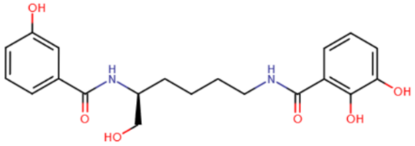	55
2-hydroxy-N-[6-[(2-hydroxybenzoyl)amino]hexyl]benzamide	Oc1ccccc1C(=O)NCCCCCCNC(=O)c2ccccc2O	79.58	ChEMBL	antibacterial activity	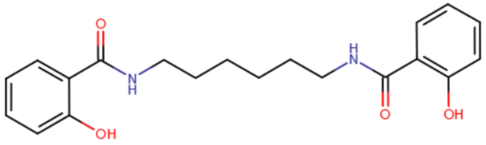	42
SP9 N-{(1R)-3-[(4-aminobutyl)amino]-1-methylpropyl}benzamide	NCCCCNCC[C@H](NC(=O)c1ccccc1)C	76.8	SwissSimilarity-Ligands from the PDB		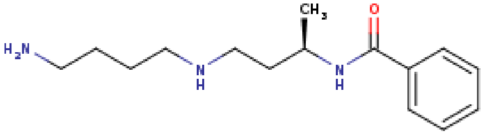	PDB:3CNT
SP8 N-{(1S)-3-[(4-aminobutyl)amino]-1-methylpropyl}benzamide	NCCCCNCC[C@@H](NC(=O)c1ccccc1)C	76.8	SwissSimilarity-Ligands from the PDB		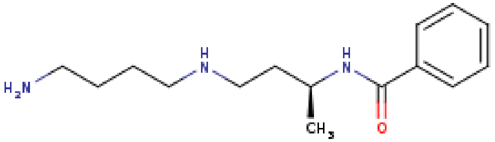	PDB:3CNS

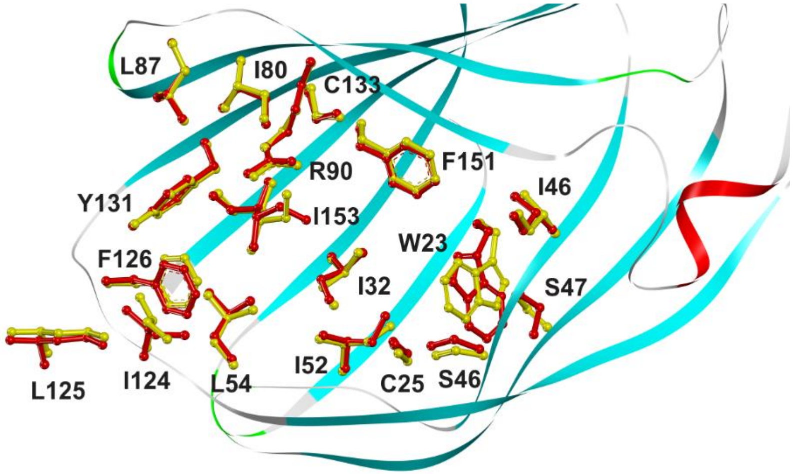

Figure S1. Superposition of MD-2-C and MD-2-D. Residues of the hydrophobic binding pocket involved in the interaction (Table 4) with the ligands are shown. Most of residues showed similar conformations or small differences, although W23, S47 and I153 showed high conformational differences. Only the side chains are shown for clarity.
